# The HIV-1 Subtype B Epidemic in French Guiana and Suriname Is Driven by Ongoing Transmissions of Pandemic and Non-pandemic Lineages

**DOI:** 10.3389/fmicb.2018.01738

**Published:** 2018-07-31

**Authors:** Gonzalo Bello, Mathieu Nacher, Flavia Divino, Edith Darcissac, Daiana Mir, Vincent Lacoste

**Affiliations:** ^1^Laboratório de AIDS e Imunologia Molecular, Instituto Oswaldo Cruz, Fundação Oswaldo Cruz, Rio de Janeiro, Brazil; ^2^Coordination Régionale de la Lutte Contre le VIH (COREVIH) and Centre d’Investigation Clinique – CIC INSERM 1424, Centre Hospitalier de Cayenne “Andrée Rosemon”, Cayenne, French Guiana; ^3^Laboratoire des Interactions Virus-Hôtes, Institut Pasteur de la Guyane, Cayenne, French Guiana

**Keywords:** HIV-1, subtype B, pandemic, non-pandemic, phylodynamics, French Guiana, Suriname

## Abstract

The HIV-1 subtype B epidemic in French Guiana and Suriname is characterized by the co-circulation of the globally disseminated “B_PANDEMIC_” lineage and of non-pandemic subtype B lineages of Caribbean origin (B_CAR_). To reconstruct the spatiotemporal pattern of spread of those viral lineages circulating in these two countries, a total of 361 HIV-1 subtype B *pol* sequences recovered from treatment-naive adult patients from French Guiana and Suriname between 2006 and 2012 were combined with B_PANDEMIC_ and B_CAR_ reference sequences. Major Guianese/Surinamese B_PANDEMIC_ and B_CAR_ lineages were identified by Maximum Likelihood phylogenetic analysis and the spatiotemporal and demographic parameters estimated using a Bayesian coalescent-based method. We detected four B_CAR_ and three B_PANDEMIC_ transmission chains of large size that together comprise most pandemic and non-pandemic subtype B sequences from French Guiana (≥52%) and Suriname (≥70%) here analyzed. These major lineages were probably introduced into French Guiana and Suriname from the Caribbean (B_CAR_) and North/South America (B_PANDEMIC_) between the middle 1970s and the late 1980s and spread among populations from both countries with roughly comparable demographic growth rates. We detected a significant trend for higher viral loads and higher proportion of homosexual/bisexual men among subjects infected with B_PANDEMIC_ relative to B_CAR_ strains in French Guiana. These results show that the HIV subtype B epidemic in French Guiana and Suriname has been driven by multiple active B_CAR_ and B_PANDEMIC_ transmission chains that arose since the middle 1970s onward and operate in both countries simultaneously. Although no significant differences in the epidemic potential of major B_CAR_ and B_PANDEMIC_ lineages were observed, relevant associations between the infecting subtype B lineage and epidemiological and clinical characteristics were detected in French Guiana.

## Introduction

The Guianas are a region located on the northeastern coast of South America, bordered by Brazil to the south and Venezuela to the west, which includes the French Guiana (an overseas department of France), and the sovereign states of Guyana (known as British Guiana until 1966) and Suriname (part of the Kingdom of the Netherlands until 1975). With a combined population of nearly 1.5 million inhabitants, the Guianas consists of a wide variety of ethnic groups due to historical colonization by Amerindians, Europeans, Africans, and Asians and recent migratory fluxes from neighboring South American and Caribbean countries (Hyles, 2014). In this singular geographic and demographic context, the HIV/AIDS epidemic is a major public health problem and HIV prevalence rates in adult populations from French Guiana, Guyana, and Suriname (1.0–1.5%) are among the highest in the American continent (Nacher et al., 2010; UNAIDS, 2013).

Subtype B is the predominant HIV-1 lineage circulating in French Guiana (Kazanji et al., 2001; Darcissac et al., 2016) and Suriname (Abdoel Wahid et al., 2016); but in sharp contrast to other continental American countries where the epidemic is mostly driven by the globally disseminated “B_PANDEMIC_” lineage, the subtype B epidemic in French Guiana and Suriname is driven by transmission of both B_PANDEMIC_ and of non-pandemic subtype B lineages characteristic of the Caribbean region (“B_CAR_” lineages) (Cabello et al., 2015). This epidemiological pattern resembles that described in several Caribbean islands (Haiti, the Dominican Republic, Jamaica, The Bahamas and the Lesser Antilles) (Cabello et al., 2014) and in the Northern Brazilian state of Roraima (Divino et al., 2016). Previous phylogenetic analyses revealed that a substantial fraction (30–95%) of subtype B infections in Latin American and Caribbean countries resulted from the expansion of a few local (or regional) B_PANDEMIC_ and B_CAR_ founder strains (Delatorre and Bello, 2013; Cabello et al., 2014, 2015; Mendoza et al., 2014; Mir et al., 2015; Divino et al., 2016), thus supporting a great geographic compartmentalization of the HIV-1 subtype B epidemic in those regions.

Little is known about the spatiotemporal dynamics of dissemination, geographic compartmentalization, and demographic history of the B_PANDEMIC_ and B_CAR_ lineages circulating in French Guiana and Suriname. To answer these questions, we used Maximum Likelihood (ML) and Bayesian coalescent-based methods to analyze a comprehensive data set of 361 HIV-1 subtype B *pol* sequences from French Guiana and Suriname recently described (Abdoel Wahid et al., 2016; Darcissac et al., 2016). The sequences were compared with HIV-1 subtype B *pol* sequences from the Caribbean, South America, North America, and Europe to identify country-specific transmission clusters of the B_PANDEMIC_ and B_CAR_ lineages and to reconstruct their evolutionary and demographic dynamics. We also tested if individuals from French Guiana infected by the B_PANDEMIC_ and B_CAR_ lineages displayed or not comparable epidemiological and clinical characteristics.

## Materials and Methods

### Guianese and Surinamese HIV-1 Subtype B Sequences

HIV-1 subtype B *pol* sequences from treatment-naive adult patients from French Guiana (*n* = 271) and Suriname (*n* = 90) recently described (Abdoel Wahid et al., 2016; Darcissac et al., 2016) were included in the present study. HIV-1 sequences were sampled over a time period of seven years (2006–2012) and cover the complete protease (PR) and the first part of the reverse transcriptase (RT) regions (nucleotides 2,253–3,275 of reference strain HXB2). Only one sequence per subject was selected and the subtype of all sequences was confirmed using the REGA HIV subtyping tool v.2 (de Oliveira et al., 2005). HIV-1 *pol* sequences were aligned using the ClustalW program (Thompson et al., 1997) and codons associated with major antiretroviral (ARV) drug resistance positions in PR (*n* = 12) and RT (*n* = 21) were excluded. All patients were informed of the possible use of epidemiological and clinical data for research and provided written consent. The project was approved by the Comité de Recherche Clinique (CoRC) Pasteur Institute Paris Project number 2014–2016.

### HIV-1 Subtype B Lineage Assignment and Identification of Guianese/Surinamese Subtype B Lineages

HIV-1 subtype B *pol* sequences from French Guiana and Suriname were first aligned with 500 subtype B sequences representative of the B_PANDEMIC_ and the B_CAR_ lineages described previously (Cabello et al., 2014; Mendoza et al., 2014) (**Supplementary Table S1**) and classified within corresponding lineages by using a ML phylogenetic approach. ML trees were inferred with the PhyML program (Guindon et al., 2010) using an online web server (Guindon et al., 2005) under the GTR+I+Γ nucleotide substitution model, as selected by the jModelTest program (Posada, 2008), and the SPR branch-swapping algorithm of heuristic tree search. The reliability of the obtained tree topology was estimated with the approximate likelihood-ratio test (*aLRT*) (Anisimova and Gascuel, 2006) based on the Shimodaira-Hasegawa-like procedure. Trees were rooted using subtype D sequences (the closets HIV-1 group M lineage relative to subtype B) taken from the Los Alamos HIV Database and visualized using the FigTree v1.4.0 program (Rambaut, 2009).

HIV-1 B_CAR_ and B_PANDEMIC_ *pol* sequences from French Guiana and Suriname were next aligned with B_CAR_ sequences from the Caribbean and Brazil and with B_PANDEMIC_ sequences from the United States (US), France, the Netherlands, and Northern Brazil characterized previously (Cabello et al., 2014, 2015, 2016; Divino et al., 2016) (**Supplementary Table S2**). Sequences were subjected to ML analyses as described above and Guianese and Surinamese B_CAR_ and B_PANDEMIC_ transmission clusters were defined as highly supported (*aLRT* ≥ 0.85) monophyletic clusters mostly (>90%) composed by sequences from these countries. For putative intra-subtype B_CAR_/B_PANDEMIC_ recombinant sequences, similarity plots depicting the percentage identity to a panel of B_PANDEMIC_, B_CAR_, and subtype D reference sequences were generated using SimPlot v.3.5.1 (Lole et al., 1999) and Neighbor-Joining phylogenetic trees of different *pol* gene fragments were reconstructed under the Tamura-Nei model, in 500 bootstrapped datasets, using MEGA v6 (Tamura et al., 2013).

### Spatiotemporal and Demographic Reconstructions

To reconstruct spatiotemporal dynamics and identify the most probable source location of major B_CAR_ Guianese/Surinamese lineages here identified, we selected B_CAR_ sequences from the major Caribbean islands with high prevalence of non-pandemic strains and B_CAR_ sequences from neighboring South American countries, including: all B_CAR_ sequences from Hispaniola and sequences of major B_CAR_ lineages circulating in Trinidad and Tobago (B_CAR-TT_), Jamaica (B_CAR-JM-I_), Brazil (B_CAR-BR-I_ and B_CAR-BR-II_), and Guyana (B_CAR-GY_) identified in previous studies (Cabello et al., 2014, 2015; Divino et al., 2016) (**Supplementary Table S3**). Similarly, to identify the most probable source location of major B_PANDEMIC_ Guianese/Surinamese lineages we selected a subset of B_PANDEMIC_ reference sequences from regions with the highest human flux from/to French Guiana and Suriname (the Caribbean, South America, Central America, North America, and Europe). A total of 40 B_PANDEMIC_ reference sequences from each geographic region with known date of isolation and with the highest similarity to Guianese and Surinamese sequences were selected using the basic local alignment search tool (BLAST)^1^ (**Supplementary Table S4**).

The evolutionary rate, the age of the most recent common ancestor (*T*_MRCA,_ years), the spatial diffusion pattern and the rate (*r*, years^-1^) of population growth of major HIV-1 Guianese/Surinamese subtype B lineages were jointly estimated using the Bayesian Markov Chain Monte Carlo (MCMC) approach as implemented in BEAST v1.8 (Drummond et al., 2002; Drummond and Rambaut, 2007) with BEAGLE (Suchard and Rambaut, 2009) to improve run-time. Because regression analyses using program TempEst (Rambaut et al., 2016) revealed that subtype B *pol* datasets here compiled does not contain sufficient temporal signal for reliable time-scale estimations (X-intercept [TMRCA] < 1,910), Bayesian MCMC analyses were performed using the GTR+I+Ã_4_ nucleotide substitution model and a relaxed uncorrelated lognormal molecular clock model (Drummond et al., 2006) with a uniform prior distribution on the substitution rate that encompass mean values previously estimated for the subtype B *pol* gene (2.0–3.0 × 10^-3^ subst./site/year) (Hue et al., 2005; Zehender et al., 2010; Chen et al., 2011; Mendoza et al., 2014). Migration events throughout the phylogenetic histories were reconstructed using a reversible discrete phylogeography model (Lemey et al., 2009) with a CTMC rate reference prior (Ferreira and Suchard, 2008). Changes in effective population size through time for each major HIV-1 Guianese/Surinamese subtype B lineages was independently estimated using a Bayesian Skyline coalescent tree prior (Drummond et al., 2005). Estimates of the population growth rate were obtained using the parametric model (logistic, exponential, or expansion) that provided the best fit to the demographic signal contained in datasets. Comparison between demographic models was performed using the log marginal likelihood estimation based on path sampling (PS) and stepping-stone sampling (SS) methods (Baele et al., 2012). MCMC chains were run for 50–200 × 10^6^ generations. Convergence (Effective Sample Size > 200) and uncertainty (95% Highest Probability Density [HPD] values) in parameter estimates were assessed using the TRACER v1.6 program (Rambaut and Drummond, 2007). Maximum clade credibility (MCC) trees were summarized with TreeAnnotator v1.7.5 and visualized with FigTree v1.4.0.

### Estimation of HIV Incidence Temporal Trend in French Guiana

To estimate the HIV incidence in French Guiana we used the Spectrum v5.51 package^2^. AIM (AIDS Impact Model) and CSAVER (Case Surveillance and Vital Registration) incidence fitting tools were used with a start in 1970 and projections until 2013. Historical HIV programmed data, treatment eligibility criteria for adults and children for different periods, the proportion of pregnant women with access to prevention of mother-to-child transmission of HIV, the number of patients receiving ARV therapy, the median CD4-count at ARV initiation, the proportion of virologically suppressed treated patients and the proportion of lost to follow-up patients each year were entered in Spectrum. The Epidemic was modeled as a concentrated epidemic. The yearly progression rate to the next CD4 category and the HIV mortality with and without ARV were selected from the options in Spectrum based on Latin America and the Caribbean. The default ratio of fertility of infected women versus uninfected ones was used.

### Statistical Analyses

Epidemiological and demographic characteristics of the cohort included in the present study were compared using Fisher’s exact test or chi2 implemented in Stata 13 software. Statistical significance was defined as *p* < 0.05.

## Results

### Identification of Major Guianese/Surinamese Subtype B Lineages

HIV-1 subtype B sequences from French Guiana and Suriname (*n* = 361) were combined with viral strains representative of the B_PANDEMIC_ (*n* = 300) and B_CAR_ (*n* = 200) lineages (**Supplementary Table S1**). The ML analysis revealed that, as expected, a very large proportion of HIV-1 subtype B sequences from French Guiana (60%) and Suriname (50%) were intermixed among B_CAR_ strains; whereas the others branched within the well-supported (*aLRT* = 0.90) B_PANDEMIC_ lineage (**Supplementary Figure S1** and **Supplementary Table S5**). A few sequences from French Guiana (4%) and Suriname (6%) remained unclassified as they branched basal to the B_PANDEMIC_ lineage, but were not intermixed among B_CAR_ sequences and, when included, greatly reduced the support of the B_PANDEMIC_ lineage (*aLRT* < 0.80) (data not shown). Similarity plots revealed that most unclassified sequences displayed a higher similarity to B_CAR_ reference strains in the initial portion of the *pol* fragment (covering the entire PR) compared to B_PANDEMIC_ strains, and a higher similarity to B_PANDEMIC_ sequences in the final portion of the *pol* fragment (**Supplementary Figure S2**). This suggests that they may represent intra-subtype B_CAR_/B_PANDEMIC_ recombinant sequences. The phylogenetic branching pattern at the initial and final portions of the *pol* fragment also supports a B_CAR_/B_PANDEMIC_ recombinant structure for half of unclassified sequences (**Supplementary Figure S2)**. However, this result should be interpreted with caution due the overall low support (bootstrap < 60%) at nodes of phylogenetic trees.

**FIGURE 1:**
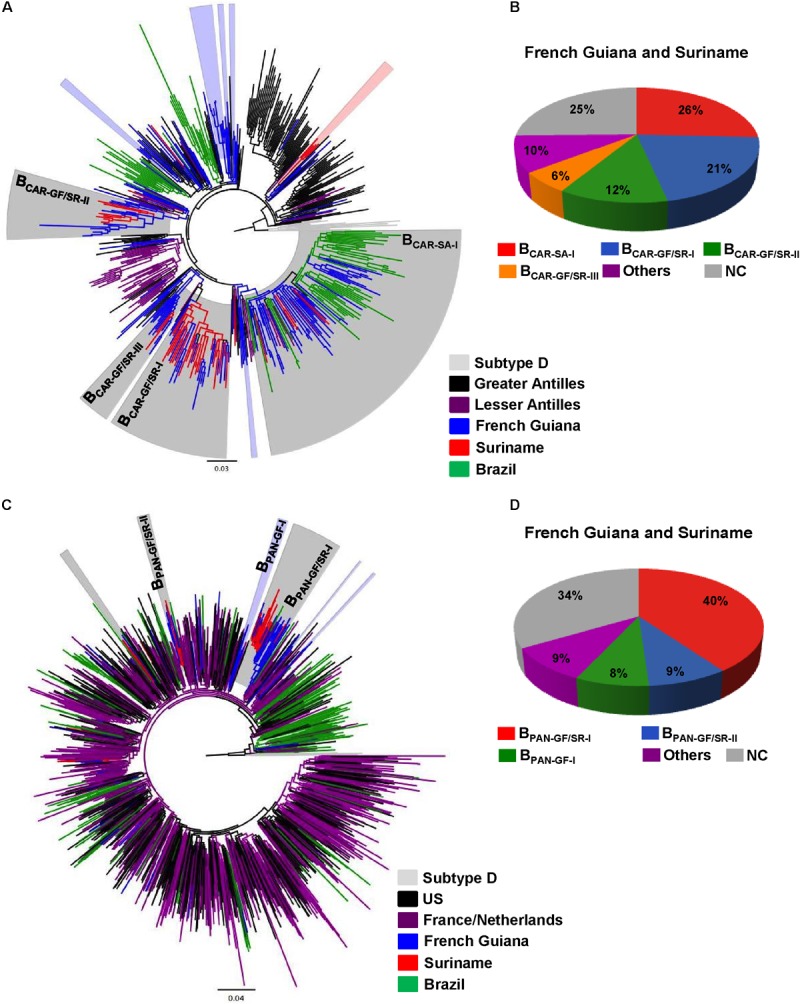
Identification of major Guianese/Surinamese subtype B lineages. **(A,C)** ML phylogenetic trees of HIV-1 B_CAR_ and B_PANDEMIC_ *pol* sequences circulating in French Guiana and Suriname together with representative B_CAR_ sequences from the Caribbean and Brazil and B_PANDEMIC_ sequences from the US, France, the Netherlands and Northern Brazil. Branches are colored according to the geographic origin of each sequence as indicated at the legend (bottom right). Shaded boxes highlight the position of B_CAR_ and B_PANDEMIC_ highly supported (SH-*aLRT* ≥ 0.85) clusters mostly/exclusively composed by Guianese and/or Surinamese sequences. Major (*n* ≥ 10) B_CAR_ and B_PANDEMIC_ lineages detected in French Guiana and Suriname are indicated with names. Trees were rooted using HIV-1 subtype D reference sequences. The branch lengths are drawn to scale with the bar at the bottom indicating nucleotide substitutions per site. **(B,D)** Estimated proportion of HIV-1 sequences branching in major and minor Guianese/Surinamese clusters as well of non-clustered (NC) sequences among B_CAR_ and B_PANDEMIC_ infected individuals from French Guiana and Suriname according to the ML analyses.

The B_CAR_ and B_PANDEMIC_ sequences from French Guiana and Suriname were then combined with B_CAR_ and B_PANDEMIC_ sequences of countries from the Americas (Caribbean region and Brazil) and Europe (France and the Netherlands) that maintain historical intense migratory fluxes with French Guiana and Suriname. The ML phylogenetic analyses revealed that most B_CAR_ and B_PANDEMIC_ sequences from French Guiana (≥52%) and Suriname (≥70%) branched within a few monophyletic lineages of large size (*n* > 10 sequences) that were exclusively composed of sequences from both countries (B_CAR-GF/SR-I_, B_CAR-GF/SR-II_, B_CAR-GF/SR-III_, B_PAN-GF/SR-I_, and B_PAN-GF/SR-II_), from French Guiana (B_PAN-GF-I_), or that also include sequences from neighboring countries (B_CAR-SA-I_) (**Figure 1** and **Supplementary Table S6**). Geographic distribution of major Guianese/Surinamese B_CAR_ and B_PANDEMIC_ lineages was not homogenous across countries (**Supplementary Tables S7, S8)**. The remaining B_CAR_ and B_PANDEMIC_ sequences from French Guiana and Suriname branched in country-specific lineages of small size (≤10%) or appeared as non-clustered infections (≤41%) (**Supplementary Table S6**).

### Spatiotemporal Dissemination of Major Guianese/Surinamese Subtype B Lineages

**FIGURE 2:**
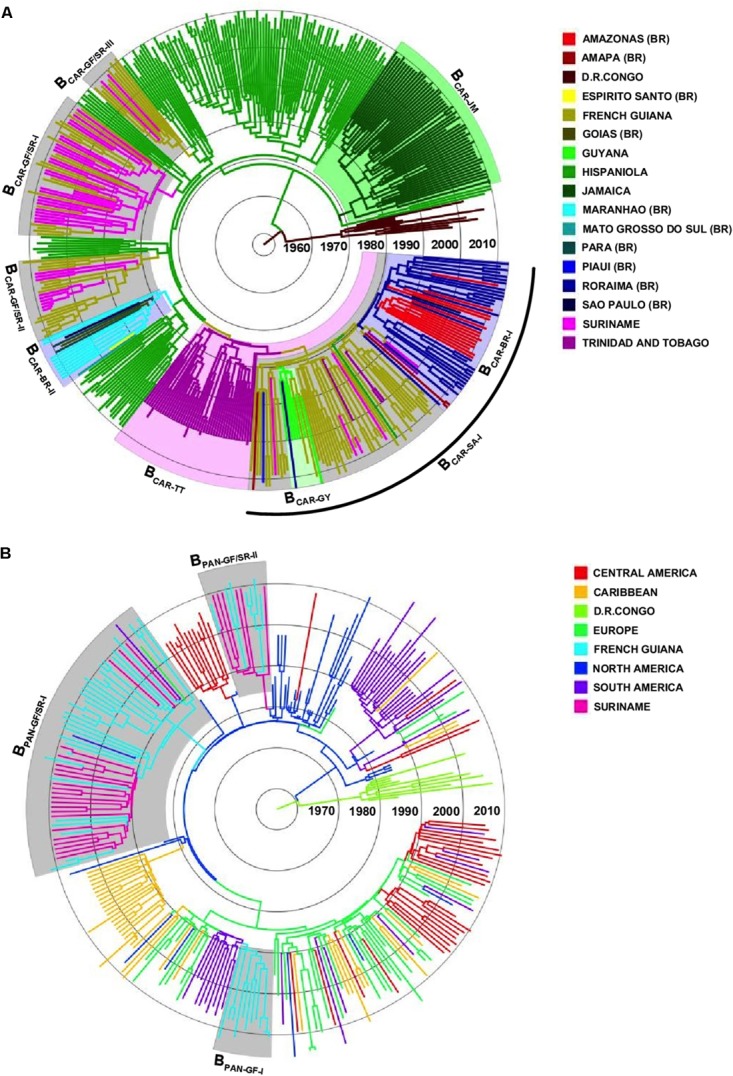
Spatiotemporal dissemination of major Guianese/Surinamese subtype B lineages. **(A)** Time-scaled Bayesian phylogenetic tree of HIV-1 B_CAR_ *pol* sequences from French Guiana, Suriname, Brazil, Guyana, and the Caribbean combined with subtype D reference sequences. Shaded boxes highlight the position of major B_CAR_ lineages from French Guiana/Suriname, Trinidad and Tobago, Jamaica, Brazil, and Guyana. The arc indicates the position of the B_CAR-SA-I_ lineage. **(B)** Time-scaled Bayesian phylogenetic tree of HIV-1 B_PANDEMIC_ *pol* sequences from the French Guiana, Suriname and closely related B_PANDEMIC_ sequences from the Caribbean, South/Central/North America, and Europe, combined with subtype D reference sequences. Shaded boxes highlight the position of major B_PANDEMIC_ lineages circulating in French Guiana and Suriname. Branches are colored according to the most probable location state of their descendent nodes as indicated in the legend at right. Branch lengths are drawn to a scale of years. The trees are automatically rooted under the assumption of a relaxed molecular clock.

To identify the most probable source location of major B_CAR_ and B_PANDEMIC_ Guianese/Surinamese subtype B lineages, those sequences were combined with sequences of major B_CAR_ lineages circulating in the Caribbean and Brazil and with B_PANDEMIC_ sequences from the Americas and Europe with the highest BLAST search similarity score to Guianese/Surinamese sequences. Bayesian phylogeographic analyses support that lineage B_CAR-SA-I_ arose after dissemination of a single variant strain from Trinidad and Tobago (*Posterior State Probability* [*PSP*] = 0.98) into French Guiana (*PSP* = 0.93) at around the middle 1970s, that subsequently passed the B_CAR-SA-I_ lineage to Suriname, Brazil, and Guyana at multiples times, originating the sublineages B_CAR-BR-I_ and B_CAR-GY_ (**Figure 2A** and **Supplementary Figure S3**). The other Guianese/Surinamese B_CAR_ lineages most probably arose after dissemination of viral strains from Hispaniola (*PSP* ≥ 0.97) into Suriname (lineage B_CAR-GF/SR-I_, *PSP* = 0.68) and French Guiana (lineages B_CAR-GF/SR-II_ and B_CAR-GF/SR-III_, *PSP* ≥ 0.97) between the late 1970s and the middle 1980s, followed by multiple viral exchanges between those countries (**Figure 2A** and **Table 1**). The lineages B_PAN-GF/SR-I_ and B_PAN-GF/SR-II_ most probably arose after concurrent dissemination of B_PANDEMIC_ strains from North America (*PSP* = 1) into French Guiana (*PSP* = 0.97) and Suriname (*PSP* = 0.75), respectively, at around the middle 1980s and were later disseminated between French Guiana and Suriname at multiple times (**Figure 2B** and **Table 1**). We detected only sporadic disseminations of the B_PAN-GF/SR-I_ lineage from French Guiana into Northern Brazil (Amapá), North America (the US), and Europe (the Netherlands). The B_PAN-GF-I_ lineage most probably arose after spreading of a B_PANDEMIC_ strain from South America (*PSP* = 0.92) into French Guiana (*PSP* = 0.99) at around the early 1990s, with no evidence of dissemination outside this country (**Figure 2B** and **Table 1**).

**Table 1:** Phylogeographic, evolutionary, and demographic parameters estimated for major HIV-1 B_CAR_ and B_PANDEMIC_ lineages circulating in French Guiana and Suriname.

Clade	*N*	Sampling interval	Origin (*PSP*)	T_MRCA_ (95% HPD)	Growth model	Growth rate (95% HPD)	R_0_ (95% HPD)
B_CAR-SA-I_	54	2000–2012	GF (0.93)	1977 (1973–1981)	Logistic	0.46 (0.30–0.64)	4.7 (3.4–6.1)
B_CAR-GF/SR-I_	45	2000–2012	SR (0.68)	1978 (1974–1982)	Logistic	0.30 (0.21–0.40)	3.4 (2.7–4.2)
B_CAR-GF/SR-II_	25	2007–2012	GF (0.97)	1980 (1975–1985)	-	-	-
B_CAR-GF/SR-III_	12	2007–2011	GF (1.00)	1984 (1979–1988)	-	-	-
B_PAN-GF/SR-I_	55	2007–2012	GF (0.97)	1985 (1982–1988)	Logistic	0.45 (0.27–0.70)	4.6 (3.2–6.6)
B_PAN-GF/SR-II_	13	2007–2011	SR (0.75)	1987 (1983–1990)	-	-	-
B_PAN-GF-I_	11	2006–2011	GF (0.99)	1990 (1987–1992)	-	-	-


*GF, French Guiana; SR, Suriname.*

### Demographic History of Major Guianese/Surinamese Subtype B Lineages

**FIGURE 3:**
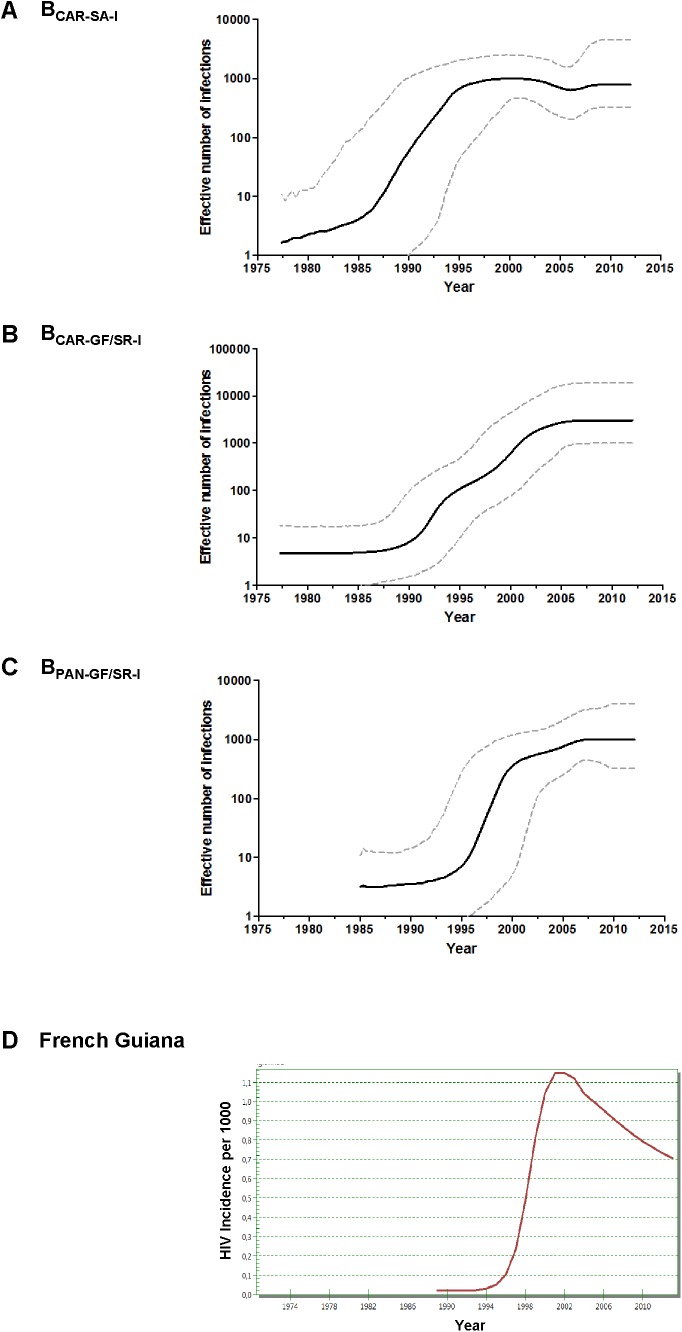
**(A–C)** Demographic history of major HIV-1 B_CAR_ and B_PANDEMIC_ lineages (*n* > 30 sequences) circulating in French Guiana and Suriname. Mean estimates of the effective number of infections (*Ne*) (solid line) are shown together with the 95% HPD intervals (dashed curves) of the Bayesian skyline plot for lineages B_CAR-SA-I_ **(A)**, B_CAR-GF/SR-I_ **(B)**, and B_PAN-GF/SR-I_ **(C)**. The vertical axes represent the estimated *Ne* on a logarithmic scale. Time scales are in calendar years. **(D)** Spectrum-estimated national HIV incidence temporal trend in French Guiana.

Bayesian coalescent inference was next used to reconstruct the population dynamics of major HIV-1 subtype B Guianese/Surinamese lineages with more than 30 sequences. The Bayesian skyline plot (BSP) analyses suggest that lineages B_CAR-SA-I_, B_CAR-GF/SR-I_, and B_PAN-GF/SR-I_ experienced an initial phase of slow growth until 1985–1990, followed by a phase of exponential growth during the 1990s and subsequent decline in growth rate from the middle 1990s and the middle 2000s (**Figures 3A–C**). The estimated temporal change of the HIV incidence rate in French Guiana also supports an epidemic stabilization around the early 2000s; but points to a continuous reduction of the HIV incidence afterward that was not captured by our coalescent-based demographic inference (**Figure 3D**). The logistic demographic model fit the data better than the other models tested (log BF > 3) for lineages B_CAR-SA-I_ and B_CAR-GF/SR-I_; but was modestly supported (log BF = 1.9) over the exponential one for lineage B_PAN-GF/SR-I_, probably due to the very recent stabilization phase (**Supplementary Table S9**). According to the logistic growth model, the mean estimated epidemic growth rates of lineages B_CAR-SA-I_ (0.46 year^-1^), B_PAN-GF/SR-I_ (0.45 year^-1^), and B_CAR-GF/SR-I_ (0.30 year^-1^) were roughly comparable and corresponded to mean R_0_ of between 3.4 and 4.7 (**Table 1**).

### Association Between Epidemiological Characteristics and HIV-1 Subtype B Lineages

Analysis of the epidemiological characteristics of Guianese subjects infected with B_PANDEMIC_ and B_CAR_ lineages revealed that both viral lineages circulated among males and females of different age groups. Most subjects infected with both B_PANDEMIC_ and B_CAR_ lineages were mainly followed-up at clinics from the capital city Cayenne and born outside French Guiana (**Table 2**). Although heterosexual sex was the main mode of HIV transmission for both lineages, the proportion of homosexual/bisexual men among those infected by the B_PANDEMIC_ lineage (10%) was five-times higher than among those infected by B_CAR_ lineages (2%). Among clinical characteristics, significant differences were observed in subject distribution according to plasma RNA viral load. The proportion of subjects with high viral loads (>10,000 copies/ml) among those infected with the B_PANDEMIC_ lineage (64%) was higher that among those infected with B_CAR_ lineages (46%).

**Table 2:** Epidemiological information of subjects from French Guiana infected by HIV-1 B_CAR_ and B_PANDEMIC_ clades.

Characteristic	Total (*n* = 260)	B_CAR_ (*n* = 162)	B_PANDEMIC_ (*n* = 98)	*P*
**Sampling interval (years)**	2007–2012	2007–2012	2007–2012	-
**HIV diagnosis**^∗^				
1980–1999	17 (7%)	10 (6%)	7 (7%)	0.67
2000–2005	35 (13%)	25 (15%)	10 (10%)	
2006–2012	202 (78%)	123 (76%)	79 (81%)	
Unknown	6 (3%)	4 (2%)	2 (2%)	
**Age group (years)**^∗∗^				
18–24	23 (9%)	14 (9%)	9 (9%)	0.55
25–34	89 (34%)	56 (35%)	33 (34%)	
35–44	72 (28%)	49 (30%)	23 (23%)	
>44	76 (29%)	43 (26%)	33 (34%)	
**Sex**^∗∗^				
Male	113 (43%)	65 (40%)	48 (49%)	0.16
Female	147 (57%)	97 (60%)	50 (51%)	
**Mode of transmission**^∗^				
Homosexual/Bisexual	14 (5%)	4 (2%)	10 (10%)	**0.009**
Heterosexual	229 (88%)	150 (93%)	79 (81%)	
Others	1 (<1%)	0	1 (1%)	
Unknown	16 (6%)	8 (5%)	8 (8%)	
**Geographic location**^∗^				
Cayenne	193 (74%)	123 (76%)	70 (71%)	0.76
Saint Laurent du Maroni	54 (21%)	31 (19%)	23 (23%)	
Others	4 (2%)	3 (2%)	1 (1%)	
Unknown	9 (3%)	5 (3%)	4 (4%)	
**Country of birth**^∗∗^				
French Guiana	31 (12%)	18 (11%)	13 (13%)	0.25
Haiti	56 (22%)	32 (20%)	24 (24%)	
Suriname	52 (20%)	32 (20%)	20 (20%)	
Guyana	33 (13%)	28 (17%)	5 (5%)	
France	33 (13%)	21 (13%)	12 (12%)	
Brazil	27 (10%)	15 (9%)	12 (12%)	
Others	13 (5%)	7 (4%)	6 (6%)	
Unknown	15 (6%)	9 (6%)	6 (6%)	
**Clinical condition**^∗^				
Asymptomatic (A)	194 (75%)	119 (73%)	75 (76%)	0.57
Symptomatic (B)	24 (9%)	18 (11%)	6 (6%)	
AIDS (C)	33 (13%)	20 (12%)	13 (13%)	
Unknown	9 (3%)	5 (3%)	4 (4%)	
**Viral load (copies/ml)**^∗^				
<LD	10 (4%)	7 (4%)	3 (3%)	**0.03**
51–2,000	42 (16%)	32 (20%)	10 (10%)	
2,001–10,000	70 (27%)	48 (30%)	22 (22%)	
>10,000	138 (53%)	75 (46%)	63 (64%)	
**CD4 count (cells/ml)**^∗∗^				
350–500	133 (51%)	85 (52%)	48 (49%)	0.58
>500	127 (49%)	77 (48%)	50 (51%)	


*^∗^Fisher’s exact test. ^∗∗^Pearson’s chi2. P values < 0.05 are in bold.*

Significant differences in epidemiological and clinical variables were also observed among major Guianese/Surinamese subtype B transmission chains (**Supplementary Table S10**). In contrast to the overall pattern, most (65%) subjects infected with the lineage B_CAR-GF/SR-I_ were followed-up at clinics from Saint Laurent du Maroni, the second most populous city of French Guiana located at the border with Suriname. Significant associations were observed between infecting strain and the country of origin, including: born in Guyana and lineage B_CAR-SA-I_, born in Suriname and lineage B_CAR-GF/SR-I_, and born in mainland France and lineage B_CAR-GF/SR-II_. Notably, individuals of Haiti origin represent a large proportion (20%) of subjects from French Guiana infected with B_CAR_ strains, but a low fraction (5%) of those infected with major Guianese/Surinamese B_CAR_ lineages. We also observed that the proportion of subjects with high viral loads among those infected with lineage B_PAN-GF/SR-I_ (82%) was higher than among those infected with lineages B_CAR-SA-I_ (43%) and B_CAR-GF/SR-II_ (25%).

## Discussion

This study revealed that the HIV-1 subtype B epidemic in French Guiana and Suriname has been driven by multiple active B_CAR_ and B_PANDEMIC_ transmission chains that arose from several independent sources and operate in both countries simultaneously. Most B_CAR_ (≥59%) and B_PANDEMIC_ (≥40%) sequences from French Guiana and Suriname here analyzed branched within regional-specific monophyletic lineages that comprise sequences from both countries; while only a minor fraction of B_CAR_ (≤10%) and B_PANDEMIC_ (≤18%) sequences branched within country-specific clusters containing only sequences from a single country. This high degree of phylogenetic intermixing of HIV sequences is fully consistent with the intense cross-border population mobility between French Guiana and Suriname (Collomb and Fouck, 2016; Jaries et al., 2017) and supports the need to develop a coordinated bi-national healthcare response.

Our analyses identify four major B_CAR_ (designated as B_CAR-SA-I_ and B_CAR-GF/SR-I_ to B_CAR-GF/SR-III_) and three major B_PANDEMIC_ (designated as B_PAN-GF/SR-I_, B_PAN-GF/SR-II_, and B_PAN-GF-I_) transmission chains that together accounted for 54 and 73% of all HIV-1 subtype B sequences from French Guiana and Suriname here analyzed, respectively. This resembles the estimated proportion of HIV-1 subtype B infections that resulted from the expansion of major local (country- and regional-specific) B_PANDEMIC_ and/or B_CAR_ lineages in Argentina (31%), Brazil (31%), Mexico (37%), El Salvador (41%), Peru (51%), Jamaica (53%), Cuba (70%), Panama (77%), Honduras (91%), and Trinidad and Tobago (94%) (Delatorre and Bello, 2013; Cabello et al., 2014; Mendoza et al., 2014; Mir et al., 2015). This level of geographic compartmentalization is much higher than that observed for the subtype B epidemic in Europe where countries are highly interconnected and transcontinental migration is a significant driving force of virus dispersal (Paraskevis et al., 2009; Magiorkinis et al., 2016) and supports that subtype B transmissions in Latin America and the Caribbean are mainly occurring between individuals from the same country or neighboring countries (Junqueira et al., 2016).

The lineage B_CAR-SA-I_ was the most widely disseminated B_CAR_ lineage in French Guiana and the northern South America region. According to our Bayesian phylogeographic analysis, this regional lineage arose after dissemination of a B_CAR-TT_ strain from Trinidad and Tobago into French Guiana at around the middle 1970s and later spread to Suriname, Guyana, and Northern Brazilian region. Nevertheless, a significant proportion (10%) of immigrants residing in Trinidad and Tobago is from Guyana (Borland et al., 2004). In addition, a very high proportion (45%) of subjects infected with the lineage B_CAR-SA-I_ in French Guiana where from Guyana. At last, the intense human mobility across the Roraima border with Guyana (Pereira, 2006; Corbin, 2007) coincides with a high prevalence of the lineage B_CAR-SA-I_ in that Northern Brazilian state (Divino et al., 2016). These epidemiological data therefore suggest that lineage B_CAR-SA-I_ arose in Guyana. Nevertheless, the scarcity of HIV-1 sequences from that country seriously constrained our phylogeographic inference. The other major Guianese/Surinamese B_CAR_ lineages most probably arose in the island of Hispaniola and were introduced into French Guiana (B_CAR-GF/SR-II_ and B_CAR-GF/SR-III_) or Suriname (B_CAR-GF/SR-I_) between the late 1970s and the middle 1980s. It is interesting to note that a significant proportion (20%) of B_CAR_-infected subjects living in French Guiana were born in Haiti but individuals of Haitian origin only represent a minor fraction (5%) of lineages B_CAR-GF/SR-I_ (5%), B_CAR-GF/SR-II_ (0%), and B_CAR-GF/SR-III_ (10%). Thus, Haitian migrants may provide an epidemiological link for sporadic B_CAR_ transmissions from Haiti into French Guiana but only a minor fraction of those migrants seems to be actively participating in sustaining the local B_CAR_ transmission networks.

Our phylogeographic analysis supports that major B_PANDEMIC_ lineages most probably arose after dissemination of viral strains from North America into French Guiana (B_PAN-GF/SR-I_) and Suriname (B_PAN-GF/SR-II_) at around the middle 1980s and from South America into French Guiana (B_PAN-GF-I_) at around the early 1990s. Despite the historical ties and intense human mobility between French Guiana/mainland France and Suriname/Netherlands (Cordova Alcaraz, 2012), our analyses only support a few sporadic disseminations of B_PANDEMIC_ strains between these regions. This is consistent with very restricted HIV transmissions between Surinamese and Dutch individuals living in the Netherlands (Kramer et al., 2011) and with the extremely low prevalence of B_CAR_ strains in mainland France and the Netherlands (Cabello et al., 2016). Similarly, no intense B_PANDEMIC_ fluxes were detected between French Guiana and Northern Brazil despite the intense human mobility and the favorable social conditions for the spread of HIV in the border region between Amapá (Northern Brazil) and French Guiana (Bourdier, 2005; Soares et al., 2011; Parriault et al., 2015; Collomb and Fouck, 2016).

Although the high population mobility promote a frequent HIV flux between French Guiana and Suriname, our results support some level of geographic subdivision for some major subtype B lineages. We found that lineages B_CAR-SA-I_ and B_PAN-GF-I_ comprise a much larger proportion of sequences from French Guiana than from Suriname, have their origin traced to French Guiana and mostly comprise Guianese subjects followed-up at the capital city, Cayenne. Lineages B_CAR-GF/SR-I_ and B_PAN-GF/SR-II_, by contrast, were much more prevalent in Suriname than in French Guiana, had their origin traced to Suriname and mostly comprise subjects followed-up at Saint Laurent du Maroni, a French Guianese city located at the border with Suriname that attend a large number of patients from that country (Nacher et al., 2010; Jaries et al., 2017). We also detected a larger proportion of non-clustered B_CAR_ and B_PANDEMIC_ infections in French Guiana relative to Suriname, perhaps reflecting the greater influence of migrations and/or the larger number of sequences analyzed from French Guiana.

The study of the epidemiological characteristics of HIV-infected subjects from French Guiana revealed that B_CAR_ and B_PANDEMIC_ viruses have been disseminated between both MSM and heterosexual individuals. The MSM group has a much greater proportion of B_PANDEMIC_ viruses (71%) than the heterosexual one (34%), but the role of MSM individuals in local spread of B_PANDEMIC_ viruses greatly varied across transmission chains. While an important fraction of subjects infected with lineages B_PAN-GF/SR-II_ (14%) and B_PAN-GF-I_ (27%) were MSM; no MSM individuals were detected within the largest local B_PAN-GF/SR-I_ transmission network. Overall, a large proportion of HIV-1 infections among heterosexuals (57%) and MSM (50%) in French Guiana can be ascribed to the seven major subtype B transmission chains here detected. More importantly, the proportion of individuals within these large cluster networks increased from 40% among those with HIV diagnosis between 1990 and 2005 to 62% among those diagnosed between 2006 and 2012. These data clearly support that large transmission clusters are a major driving force sustaining the recent dissemination of B_CAR_ and B_PANDEMIC_ epidemics in French Guiana. These results also emphasize that early detection and treatment as well as Pre Exposure Prophylaxis targeting people being part of large transmission chains should have a significant impact on reducing the spread of HIV-1 in French Guiana.

The analysis of clinical characteristics of individuals from French Guiana revealed a significant trend for higher RNA viral loads among B_PANDEMIC_-infected relative to B_CAR_-infected subjects, despite no significant differences in clinical condition or CD4^+^ T cell counts between groups. Previous studies suggested that viral genetic factors contribute to the heritability and variation of viral load set point (Bonhoeffer et al., 2015; Bertels et al., 2018) and may also modulate viral replication (Ronsard et al., 2017a,b). Whether differences in viral load here observed between subjects reflect selective advantages for BPANDEMIC strains over B_CAR_ ones cannot be answered by the present study. Future *in vitro* studies comparing the replication dynamics of B_CAR_ and B_PANDEMIC_ strains and studies of carefully controlled retrospective cohorts could further explore this possibility. Characterization of full-length viral genomes should also be done in future studies to detect potential intra-subtype B_CAR_/B_PANDEMIC_ recombinant lineages.

Although major lineages B_PAN-GF/SR-I_, B_CAR-SA-I_, and B_CAR-GF/SR-I_ showed significant differences regarding city of residence, country of origin and RNA viral load, they exhibited roughly comparable mean epidemic growth rates (0.30–0.46 years^-1^). Transactional sex and concurrent sexual partnerships were pointed as important drivers of the HIV epidemic in French Guiana (Nacher et al., 2010) and most subjects (>90%) infected by major Guianese/Surinamese lineages were heterosexuals. Interestingly, the epidemic growth rates here obtained were similar to those estimated for B_CAR_ and B_PANDEMIC_ lineages spreading in countries from the Caribbean and Central America with generalized heterosexual epidemics (0.35–0.45 years^-1^) (Cabello et al., 2014; Mendoza et al., 2014); but lower than those estimated for B_PANDEMIC_ lineages mainly transmitted among MSM networks (0.75–1.55 years^-1^) (Hue et al., 2005; Zehender et al., 2010; Chen et al., 2011; Delatorre and Bello, 2013; Worobey et al., 2016). This supports that the characteristics of the existing transmission network is a major driving force of the epidemic potential of different HIV-1 subtype B lineages.

In summary, this study highlights that HIV epidemics in French Guiana and Suriname are highly intermixed and that about 60% of HIV-1 subtype B infections in those countries resulted from the expansion of multiple B_CAR_ and B_PANDEMIC_ viral strains probably introduced between the middle 1970s and the early 1990s. Major B_CAR_ and B_PANDEMIC_ local lineages have independently spread among males and females of different age and risk groups from both countries and substantially contribute to the ongoing epidemic. Some associations between the infecting B_CAR_/B_PANDEMIC_ lineage and epidemiological (geographic location and country of birth) and clinical (RNA viral load) variables were detected among individuals sampled in French Guiana, but no major differences in the epidemic potential of major B_CAR_ and B_PANDEMIC_ lineages were observed.

## Author Contributions

GB and VL conceived and designed the study. MN, ED, and VL collected and analyzed the epidemiological data. MN performed the estimation of the HIV incidence temporal trend in French Guiana. FD performed the phylogenetic analyses. DM and GB performed the phylodynamics inferences and produced all figures. GB, MN, and VL wrote the manuscript. All authors discussed and reviewed the manuscript.

## Conflict of Interest Statement

The authors declare that the research was conducted in the absence of any commercial or financial relationships that could be construed as a potential conflict of interest.

## Footnotes

1 www.ncbi.nlm.nih.gov/BLAST

2 http://www.avenirhealth.org/software-spectrum.php

## Supplementary Material

FIGURE S1 Lineage assignment of HIV-1 subtype B *pol* sequences from French Guiana and Suriname. **(A,C)** ML phylogenetic trees of HIV-1 subtype B *pol* PR/RT sequences (∼1,000 nt) from French Guiana (*n* = 271) and Suriname (*n* = 90) together with representative sequences of the B_PANDEMIC_ (US = 165 and France = 135) and the B_CAR_ (Caribbean = 200) lineages. Branches are colored according to the geographic origin of each sequence as indicated at the legend (bottom right). Asterisks point to highly supported (SH-*aLRT* > 0.90) key nodes of subtype B and the B_PANDEMIC_ lineage. Trees were rooted using HIV-1 subtype D reference sequences. The branch lengths are drawn to scale with the bar at the bottom indicating nucleotide substitutions per site. **(B,D)** Estimated proportion of B_CAR_ and B_PANDEMIC_ lineages among HIV-1 subtype B infected individuals from French Guiana and Suriname according to the ML analyses. ND, not determined.

FIGURE S2 Analysis of HIV-1 subtype B *pol* sequences with no lineage assignment. Similarity plots generated using SimPlot are shown on the left. Plots displays the percent genetic similarity (*y* axis) of each subtype B query sequence from French Guiana/Suriname to the reference B_CAR_ (blue line), B_PANDEMIC_ (red line), and subtype D (black line) strains along the *pol* gene fragment (*x* axis; nucleotides 2,253–3,275 of reference strain HXB2). The analysis was performed with a window size of 250 nucleotides (nt) and a step size of 10 nt. Dashed vertical lines delimitate the *pol* gene fragments were most query sequences displayed highest similarity to B_CAR_ references (fragment I), near equal similarity to both B_CAR_ and B_PANDEMIC_ references (fragment II), and highest similarity to B_PANDEMIC_ references (fragment III). The GenBank accession number of each sequence is indicated in the upper left. NJ phylogenetic trees of subtype B query sequences from French Guiana/Suriname combined with B_CAR_ (blue circles), B_PANDEMIC_ (red circles), and subtype D (black circles) strains at *pol* fragments I and III are shown on the left. The subtype/lineage assignment of each query sequence at each fragment is indicated in the upper middle. Subtype D sequences were used as outgroups and the branch lengths are drawn to scale with the bar at the bottom indicating nucleotide substitutions per site. Low bootstrap support values were obtained for both subtype B (<70%) and B_PANDEMIC_ (<30%) monophyletic groups in all phylogenetic trees.

FIGURE S3 Map summarizing viral migration events of major HIV-1 B_CAR_ lineages circulating in French Guiana and Suriname. Arrows between locations represent branches in the Bayesian phylogenetic tree along, which location’s transitions occur. Map was created with Quantum GIS (QGIS) software from templates obtained from d-maps.com (Caribbean: http://d-maps.com/pays.php?num_pay=118&lang=es; South America: http://d-maps.com/carte.php?num_car=28522&lang=en).

TABLE S1 HIV-1 subtype B pol (PR/RT) reference sequences of BPANDEMIC and BCAR clades used for subtype B clade assignment.

TABLE S2 HIV-1 subtype B pol (PR/RT) reference sequences of BPANDEMIC and BCAR clades used for identification of Guianese/Surinamese subtype B clades.

TABLE S3 HIV-1 BCAR pol (PR/RT) sequences used for Bayesian phylogeographic analysis.

TABLE S4 HIV-1 BPANDEMIC pol (PR/RT) sequences used for Bayesian phylogeographic analysis.

TABLE S5 Clade assignment of HIV-1 subtype B subtype pol sequences from French Guiana and Suriname.

TABLE S6 Phylogenetic clustering of HIV-1 BCAR and BPANDEMIC pol sequences from French Guiana and Suriname.

TABLE S7 Prevalence of major HIV-1 BCAR lineages circulating in French Guiana and Suriname.

TABLE S8 Prevalence of major HIV-1 BPANDEMIC lineages circulating in French Guiana and Suriname.

TABLE S9 Best fit demographic models for major HIV-1 subtype B lineages circulating in French Guiana and Suriname.

TABLE S10 Epidemiological information of subjects from French Guiana infected by major HIV-1 BCAR and BPANDEMIC lineages.
