# Supplementary figures and images for: The HIV-1 Subtype B Epidemic in French Guiana and Suriname Is Driven by Ongoing Transmissions of Pandemic and Non-pandemic Lineages
Source: Front Microbiol. 2018 Jul 31;9:1738. doi: 10.3389/fmicb.2018.01738 (PMC6079251; doi:10.3389/fmicb.2018.01738)

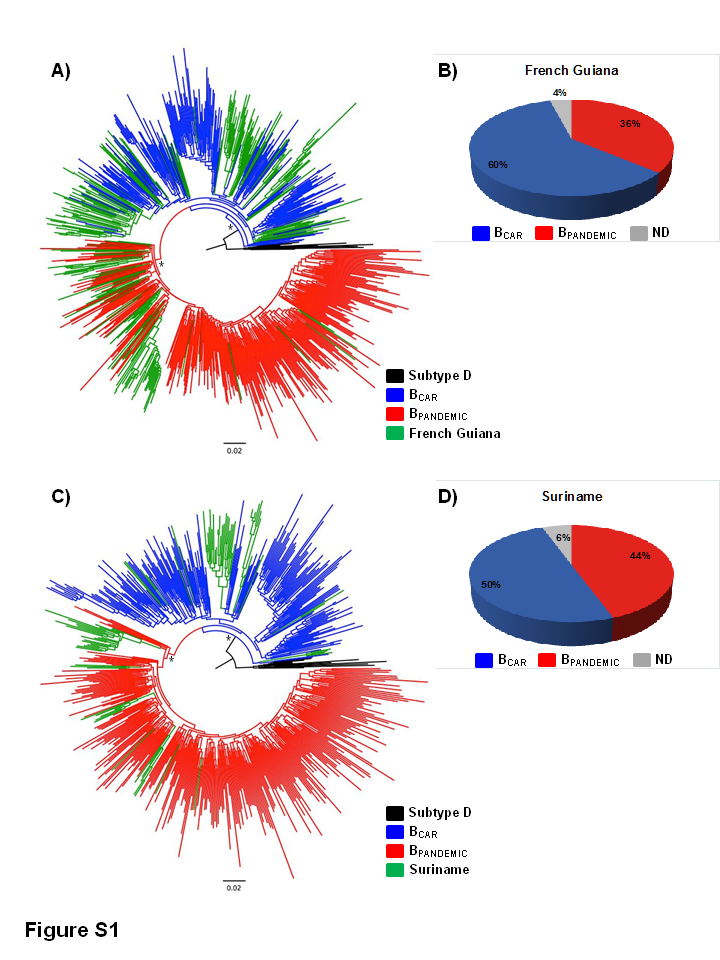

Supplement: FIGURE S1 [file Image_1.TIF]

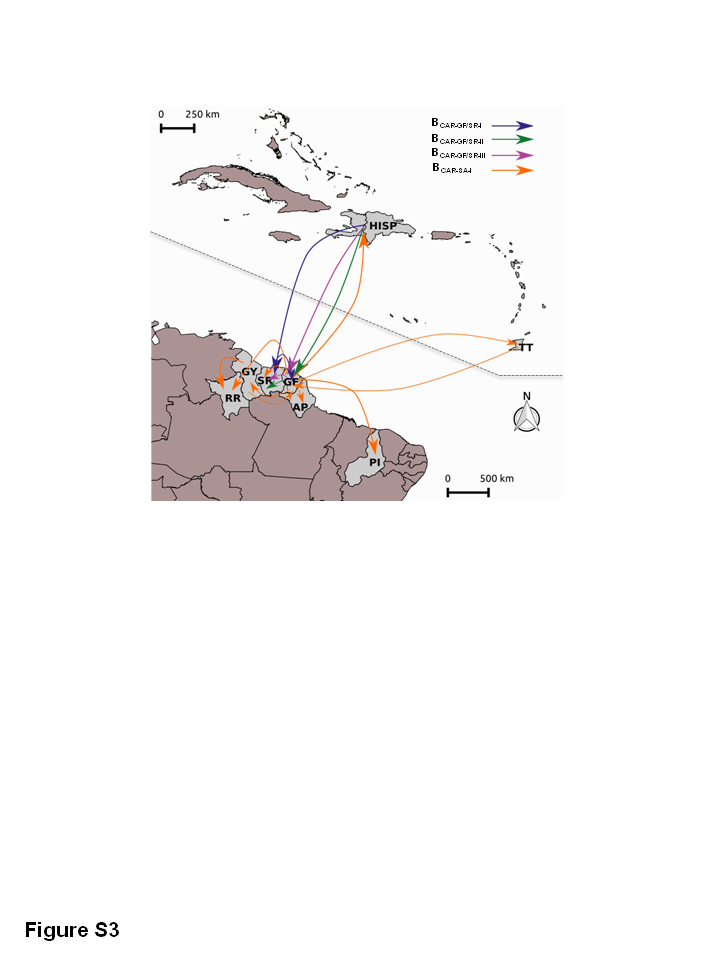

Supplement: FIGURE S3 [file Image_2.TIF]
