# Supplementary material for: The HIV-1 Subtype B Epidemic in French Guiana and Suriname Is Driven by Ongoing Transmissions of Pandemic and Non-pandemic Lineages
Source: Front Microbiol. 2018 Jul 31;9:1738. doi: 10.3389/fmicb.2018.01738 (PMC6079251; doi:10.3389/fmicb.2018.01738)
Supplement: FIGURE S2 [file Data_Sheet_1.PDF]

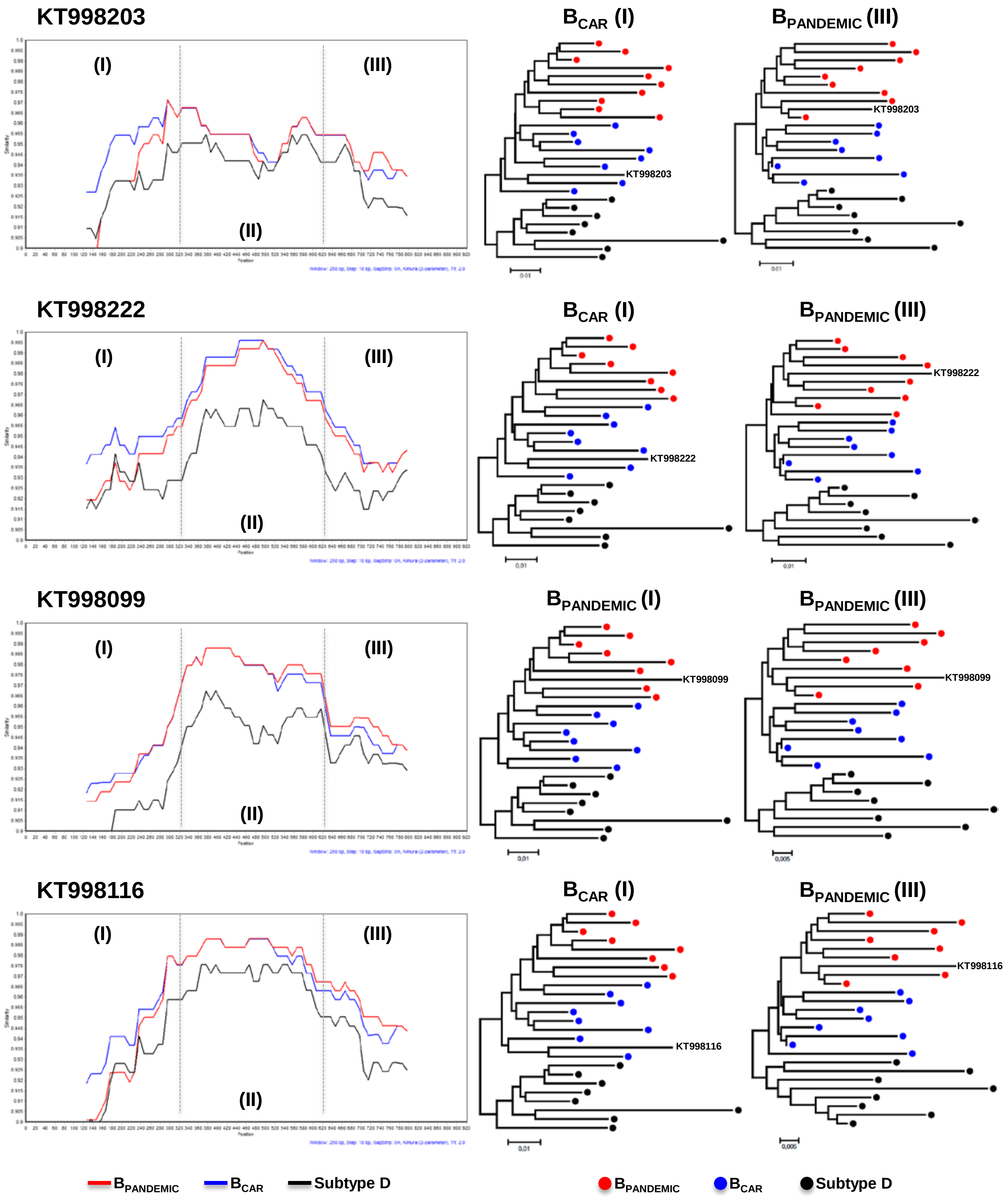
KT998203
(I)
(II)
(III)
BCAR (I)
BPANDEMIC (III)
KT998203
KT998203
KT998222
(I)
(II)
(III)
BCAR (I)
BPANDEMIC (III)
KT998222
KT998222
KT998099
(I)
(II)
(III)
BPANDEMIC (I)
BPANDEMIC (III)
KT998099
KT998099
KT998116
(I)
(II)
(III)
BCAR (I)
BPANDEMIC (III)
KT998116
KT998116
BPANDEMIC
BCAR
Subtype D
BPANDEMIC
BCAR
Subtype D

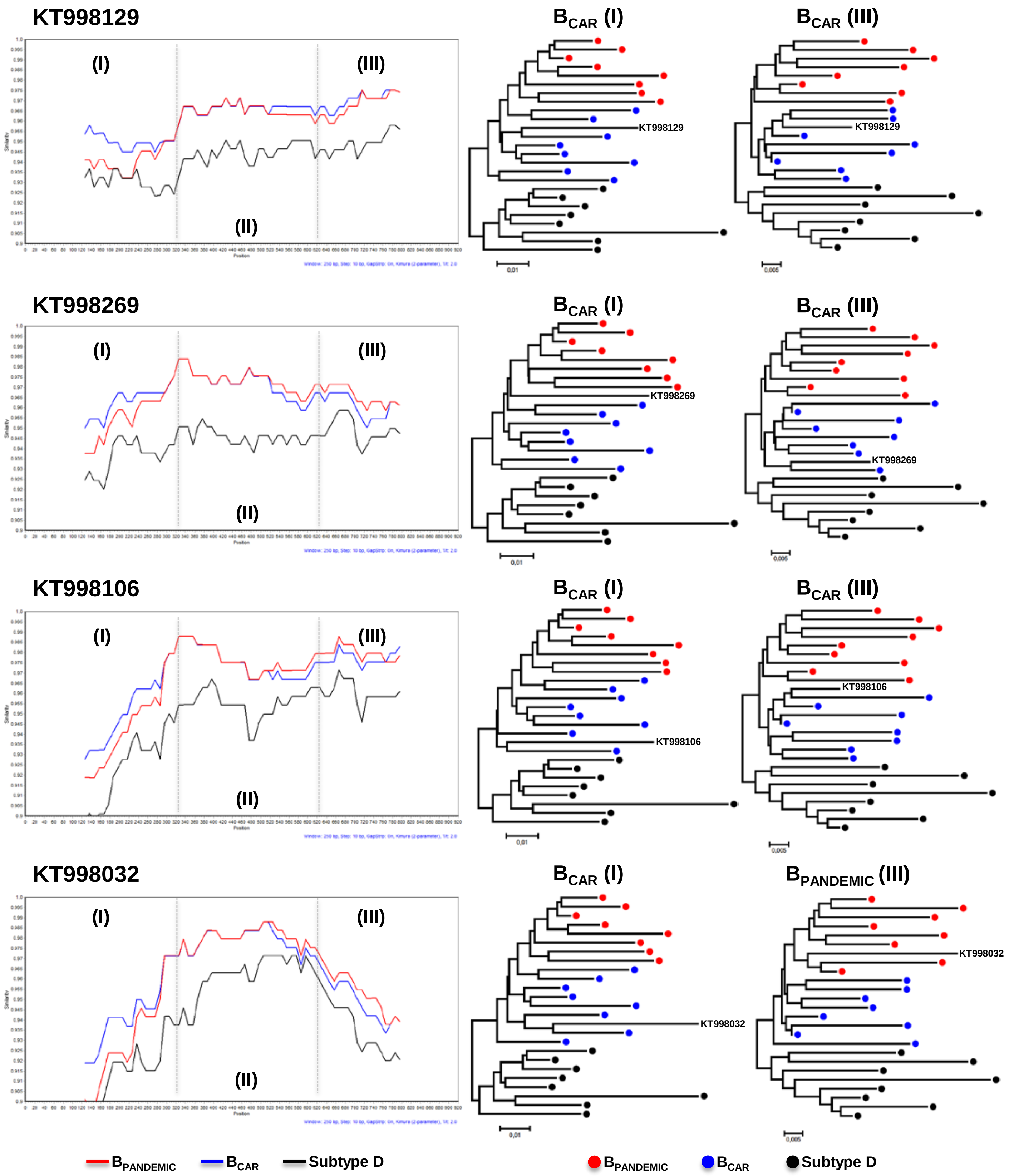
KT998129
BCAR (I)
BCAR (III)
(I)
(II)
(III)
KT998129
0,01
0,005
KT998269
BCAR (I)
BCAR (III)
(I)
(II)
(III)
KT998269
0,01
0,005
KT998106
BCAR (I)
BCAR (III)
(I)
(II)
(III)
KT998106
0,01
0,005
KT998032
BCAR (I)
BPANDEMIC (III)
(I)
(II)
(III)
KT998032
0,01
0,005
BPANDEMIC
BCAR
Subtype D
BPANDEMIC
BCAR
Subtype D

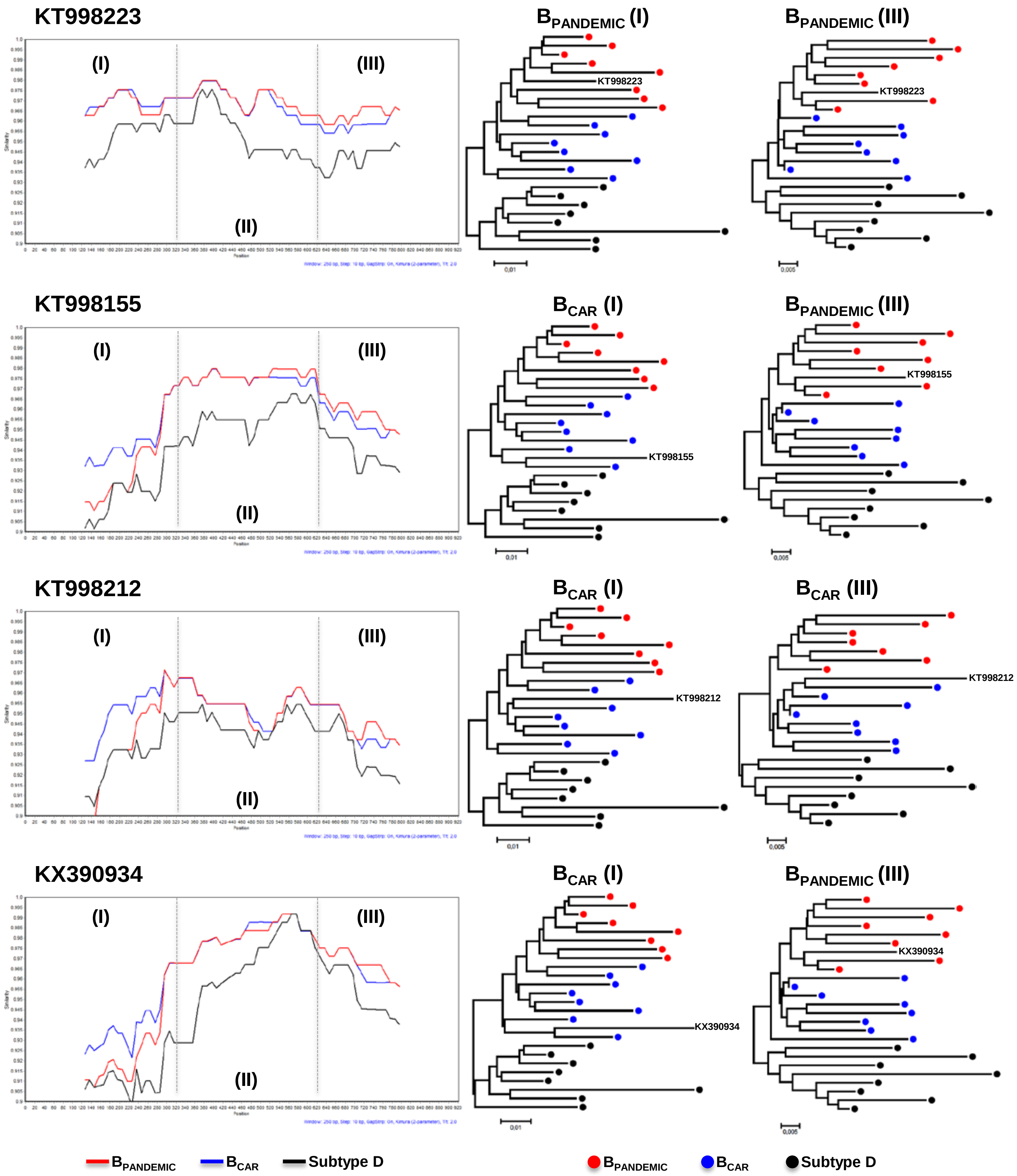
KT998223
(I)
(II)
(III)
B PANDEMIC (I)
B PANDEMIC (III)
KT998223
0,01
0,005
KT998155
(I)
(II)
(III)
B CAR (I)
B PANDEMIC (III)
KT998155
0,01
0,005
KT998212
(I)
(II)
(III)
B CAR (I)
B CAR (III)
KT998212
0,01
0,005
KX390934
(I)
(II)
(III)
B CAR (I)
B PANDEMIC (III)
KX390934
0,01
0,005
B PANDEMIC
B CAR
Subtype D
B PANDEMIC
B CAR
Subtype D

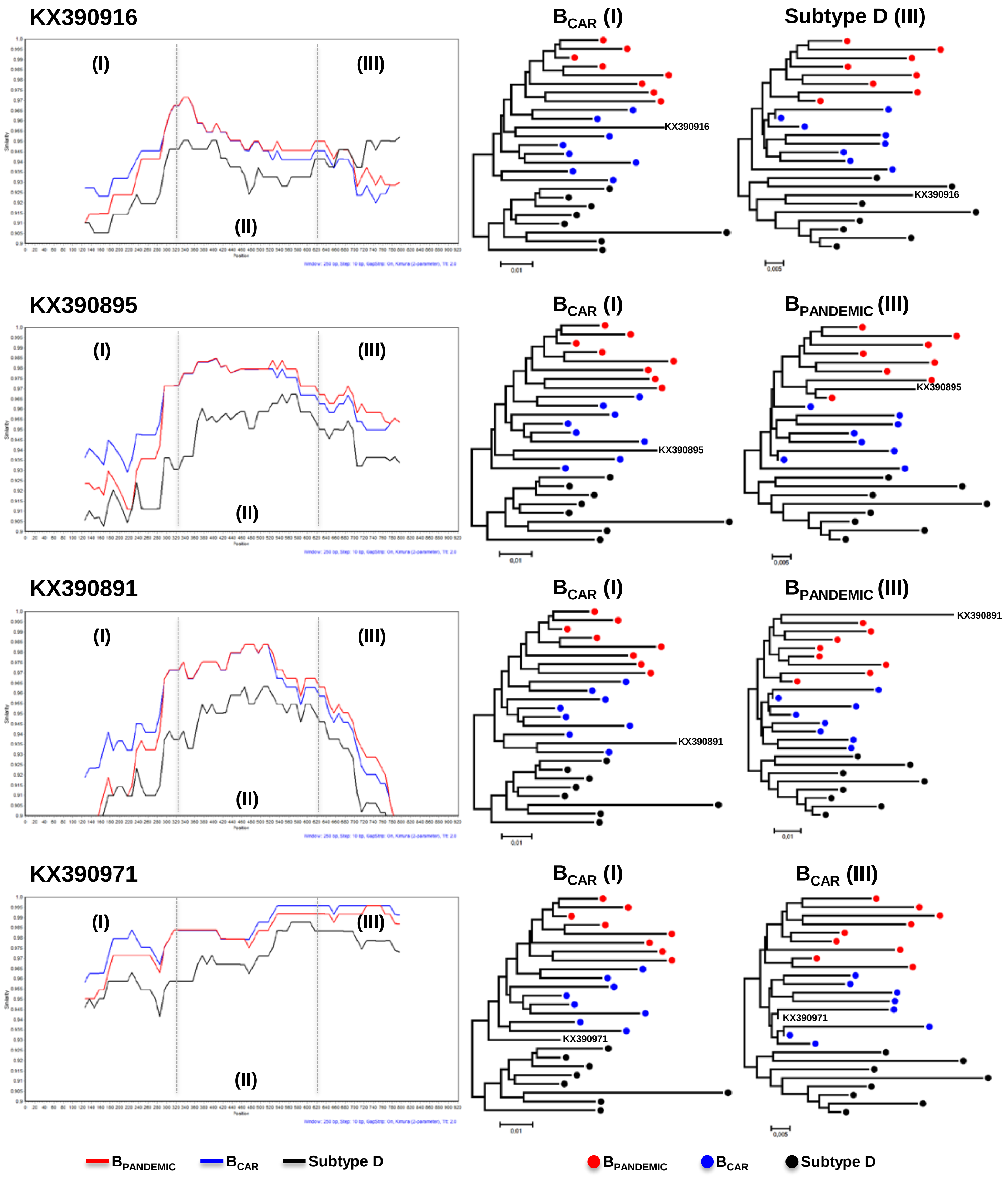
KX390916
B_{CAR} (I)
Subtype D (III)
(I)
(II)
(III)
KX390916
0.01
0.005
KX390895
B_{CAR} (I)
$B_{PANDEMIC}$ (III)
KX390895
KX390891
B_{CAR} (I)
$B_{PANDEMIC}$ (III)
KX390891
KX390971
B_{CAR} (I)
B_{CAR} (III)
KX390971
$B_{PANDEMIC}$
B_{CAR}
Subtype D
