# Supplementary material for: The HIV-1 Subtype B Epidemic in French Guiana and Suriname Is Driven by Ongoing Transmissions of Pandemic and Non-pandemic Lineages
Source: Front Microbiol. 2018 Jul 31;9:1738. doi: 10.3389/fmicb.2018.01738 (PMC6079251; doi:10.3389/fmicb.2018.01738)
Supplement: TABLE S1 [file Table_1.DOCX]

**Table 1.** Phylogeographic, evolutionary and demographic parameters estimated for major HIV-1 B_CAR_ and B_PANDEMIC_ lineages circulating in French Guiana and Suriname.

| Clade | *N* | Sampling interval | Origin (*PSP*) | T_MRCA_  (95% HPD) | Growth model | Growth rate  (95% HPD) | R_0_  (95% HPD) |
| --- | --- | --- | --- | --- | --- | --- | --- |
| B_CAR–SA-I_ | 54 | 2000-2012 | GF  (0.93) | 1977  (1973-1981) | Logistic | 0.46  (0.30-0.64) | 4.7  (3.4-6.1) |
| B_CAR–GF/SR-I_ | 45 | 2000-2012 | SR  (0.68) | 1978  (1974-1982) | Logistic | 0.30  (0.21-0.40) | 3.4  (2.7-4.2) |
| B_CAR–GF/SR-II_ | 25 | 2007-2012 | GF  (0.97) | 1980  (1975-1985) | - | - | - |
| B_CAR–GF/SR-III_ | 12 | 2007-2011 | GF  (1.00) | 1984  (1979-1988) | - | - | - |
| B_PAN–GF/SR-I_ | 55 | 2007-2012 | GF  (0.97) | 1985  (1982-1988) | Logistic | 0.45  (0.27-0.70) | 4.6  (3.2-6.6) |
| B_PAN–GF/SR-II_ | 13 | 2007-2011 | SR  (0.75) | 1987  (1983-1990) | - | - | - |
| B_PAN–GF-I_ | 11 | 2006-2011 | GF  (0.99) | 1990  (1987-1992) | - | - | - |

GF: French Guiana. SR: Suriname.
