# Supplementary material for: The HIV-1 Subtype B Epidemic in French Guiana and Suriname Is Driven by Ongoing Transmissions of Pandemic and Non-pandemic Lineages
Source: Front Microbiol. 2018 Jul 31;9:1738. doi: 10.3389/fmicb.2018.01738 (PMC6079251; doi:10.3389/fmicb.2018.01738)
Supplement: TABLE S2 [file Table_2.DOCX]

**Table 2.** Epidemiological information of subjects from French Guiana infected by HIV-1 B_CAR_ and B_PANDEMIC_ clades.

| **Characteristic** | **Total**  **(n = 260)** | **B_CAR_**  **(n = 162)** | **B_PANDEMIC_**  **(n = 98)** | ***P*** |
| --- | --- | --- | --- | --- |
| **Sampling interval (years)** | 2007-2012 | 2007-2012 | 2007-2012 | - |
| **HIV diagnosis*** |  |  |  |  |
| 1980-1999 | 17 (7%) | 10 (6%) | 7 (7%) | 0.67 |
| 2000-2005 | 35 (13%) | 25 (15%) | 10 (10%) |  |
| 2006-2012 | 202 (78%) | 123 (76%) | 79 (81%) |  |
| Unknown | 6 (3%) | 4 (2%) | 2 (2%) |  |
| **Age group (years)**** |  |  |  |  |
| 18-24 | 23 (9%) | 14 (9%) | 9 (9%) | 0.55 |
| 25-34 | 89 (34%) | 56 (35%) | 33 (34%) |  |
| 35-44 | 72 (28%) | 49 (30%) | 23 (23%) |  |
| >44 | 76 (29%) | 43 (26%) | 33 (34%) |  |
| **Sex**** |  |  |  |  |
| Male | 113 (43%) | 65 (40%) | 48 (49%) | 0.16 |
| Female | 147 (57%) | 97 (60%) | 50 (51%) |  |
| **Mode of Transmission*** |  |  |  |  |
| Homosexual/Bisexual | 14 (5%) | 4 (2%) | 10 (10%) | **0.009** |
| Heterosexual | 229 (88%) | 150 (93%) | 79 (81%) |  |
| Others | 1 (<1%) | 0 | 1 (1%) |  |
| Unknown | 16 (6%) | 8 (5%) | 8 (8%) |  |
| **Geographic location*** |  |  |  |  |
| Cayenne | 193 (74%) | 123 (76%) | 70 (71%) | 0.76 |
| Saint Laurent du Maroni | 54 (21%) | 31 (19%) | 23 (23%) |  |
| Others | 4 (2%) | 3 (2%) | 1 (1%) |  |
| Unknown | 9 (3%) | 5 (3%) | 4 (4%) |  |
| **Country of birth**** |  |  |  |  |
| French Guiana | 31 (12%) | 18 (11%) | 13 (13%) | 0.25 |
| Haiti | 56 (22%) | 32 (20%) | 24 (24%) |  |
| Suriname | 52 (20%) | 32 (20%) | 20 (20%) |  |
| Guyana | 33 (13%) | 28 (17%) | 5 (5%) |  |
| France | 33 (13%) | 21 (13%) | 12 (12%) |  |
| Brazil | 27 (10%) | 15 (9%) | 12 (12%) |  |
| Others | 13 (5%) | 7 (4%) | 6 (6%) |  |
| Unknown | 15 (6%) | 9 (6%) | 6 (6%) |  |
| **Clinical Condition*** |  |  |  |  |
| Asymptomatic (A) | 194 (75%) | 119 (73%) | 75 (76%) | 0.57 |
| Symptomatic (B) | 24 (9%) | 18 (11%) | 6 (6%) |  |
| AIDS (C) | 33 (13%) | 20 (12%) | 13 (13%) |  |
| Unknown | 9 (3%) | 5 (3%) | 4 (4%) |  |
| **Viral load (copies/ml)*** |  |  |  |  |
| <LD | 10 (4%) | 7 (4%) | 3 (3%) | **0.03** |
| 51-2,000 | 42 (16%) | 32 (20%) | 10 (10%) |  |
| 2,001-10,000 | 70 (27%) | 48 (30%) | 22 (22%) |  |
| >10,000 | 138 (53%) | 75 (46%) | 63 (64%) |  |
| **CD4 count (cells/ml)**** |  |  |  |  |
| 350-500 | 133 (51%) | 85 (52%) | 48 (49%) | 0.58 |
| >500 | 127 (49%) | 77 (48%) | 50 (51%) |  |

*Fisher’s exact test. ** Pearson’s chi2.
