# Supplementary material for: The HIV-1 Subtype B Epidemic in French Guiana and Suriname Is Driven by Ongoing Transmissions of Pandemic and Non-pandemic Lineages
Source: Front Microbiol. 2018 Jul 31;9:1738. doi: 10.3389/fmicb.2018.01738 (PMC6079251; doi:10.3389/fmicb.2018.01738)
Supplement: TABLE S3 [file Table_3.DOCX]

Supplementary Tables

**Table S1.** HIV-1 subtype B *pol* (PR/RT) reference sequences of B_PANDEMIC_ and B_CAR_ clades used for subtype B clade assignment.

| **Clade** | **Country** | ***N*** | **Sampling time** |
| --- | --- | --- | --- |
| B_PANDEMIC_ | France | 135 | 1985-2008 |
|  | US | 165 | 1997-2009 |
| B_CAR_ | Dominican Republic | 61 | 2005-2010 |
|  | Haiti | 8 | 2004-2005 |
|  | Jamaica | 62 | 2005-2010 |
|  | Trinidad and Tobago | 48 | 2000-2003 |
|  | Other Caribbean countries ^a^ | 21 | 2000-2004 |

^a^ Antigua and Barbuda (*n* = 4), Bahamas (*n* = 5), Dominica (*n* = 1), Grenada (*n* = 2), Montserrat (*n* = 1), Saint Lucia (*n* = 4) and Saint Vincent and the Grenadines (*n* = 4).

**Table S2.** HIV-1 subtype B *pol* (PR/RT) reference sequences of B_PANDEMIC_ and B_CAR_ clades used for identification of Guianese/Surinamese subtype B clades.

| **Clade** | **Country** | ***N*** | **Sampling time** |
| --- | --- | --- | --- |
| B_PANDEMIC_ | Brazil (Northern region) | 216 | 2010-2013 |
|  | France | 437 | 1983-2012 |
|  | Netherlands | 453 | 1986-2012 |
|  | US | 470 | 1982-2010 |
| B_CAR_ | Brazil | 86 | 1997-2013 |
|  | Dominican Republic | 61 | 2005-2010 |
|  | Haiti | 8 | 2004-2005 |
|  | Jamaica | 48 | 2005-2010 |
|  | Trinidad and Tobago | 48 | 2000-2003 |
|  | Other Caribbean countries ^a^ | 21 | 2000-2004 |

^a^ Antigua and Barbuda (*n* = 4), Bahamas (*n* = 5), Dominica (*n* = 1), Grenada (*n* = 2), Montserrat (*n* = 1), Saint Lucia (*n* = 4) and Saint Vincent and the Grenadines (*n* = 4).

**Table S3.** HIV-1 B_CAR_ *pol* (PR/RT) sequences used for Bayesian phylogeographic analysis.

| **Country** | **State** | **Location** | ***N*** | **Sampling date** |
| --- | --- | --- | --- | --- |
| French Guiana | - | FG | 94 | 2006-2012 |
| Suriname ^a^ | - | SR | 41 | 2000-2009 |
| Guyana | - | GY | 7 | 2000-2013 |
| Brazil | AM | AM | 14 | 2009-2011 |
|  | AP | AP | 3 | 2013 |
|  | PA | PA | 2 | 2010 |
|  | RR | RR | 32 | 2010-2013 |
|  | MA | MA | 10 | 2012 |
|  | PI | PI | 1 | 2011 |
|  | GO | GO | 1 | 2008 |
|  | MS | MS | 1 | 2008 |
|  | ES | ES | 1 | 1997 |
|  | SP | SP | 2 | 2003-2006 |
| Dominican Republic | - | HISP | 123 | 2003-2011 |
| Haiti | - |  | 12 | 2004-2005 |
| Jamaica | - | JM | 53 | 2005-2010 |
| Trinidad and Tobago | - | TT | 48 | 2000-2003 |
| DRC ^b^ | - | CD | 10 | 1983-2007 |

^a^ Includes B_CAR_ *pol* sequences sampled in 2009 (*n* = 38)^4^ and 2000 (*n* = 3)^5^. ^b^ Subtype D sequences from the Democratic Republic of Congo (DRC).

**Table S4.** HIV-1 B_PANDEMIC_ *pol* (PR/RT) sequences used for Bayesian phylogeographic analysis.

| **Region** | **Country** | **Location** | ***N*** | **Sampling date** |
| --- | --- | --- | --- | --- |
| South America | French Guiana | GF | 51 | 2006-2012 |
|  | Suriname | SR | 28 | 2009 |
|  | Argentina | SAM | 9 | 2000-2008 |
|  | Brazil |  | 26 | 1998-2015 |
|  | Peru |  | 1 | 2003 |
|  | Venezuela |  | 4 | 2005-2007 |
| Central America | Honduras | CAM | 12 | 2002-20006 |
|  | Mexico |  | 23 | 2005-2009 |
|  | Panama |  | 3 | 2005-2010 |
|  | El Salvador |  | 2 | 2008-2010 |
| North America | Canada | NA | 4 | 1984-2003 |
|  | US |  | 36 | 1978-2015 |
| Caribbean | Cuba | CAR | 35 | 2003-2012 |
|  | Dominican Republic |  | 1 | 2011 |
|  | Jamaica |  | 4 | 2009 |
| Western Europe | Denmark | EU | 2 | 2014 |
|  | France |  | 2 | 1983-2003 |
|  | Germany |  | 8 | 1997-2009 |
|  | Netherlands |  | 1 | 2006 |
|  | Spain |  | 15 | 2001-2013 |
|  | Switzerland |  | 3 | 1995 |
|  | United Kingdom |  | 9 | 1997-2010 |
| Africa | DRC | CD | 10 | 1983-2007 |

^a^ Subtype D sequences from the Democratic Republic of Congo (DRC).

**Table S5.** Clade assignment of HIV-1 subtype B subtype *pol* sequences from French Guiana and Suriname.

| **Country** | ***N*** | **B_CAR_** | **B_PANDEMIC_** | **Unclassified** | ***P**** |
| --- | --- | --- | --- | --- | --- |
| GF | 271 | 162 (60%) | 98 (36%) | 11 (4%) | 0.25 |
| SR | 90 | 45 (50%) | 40 (44%) | 5 (6%) |  |

*Fisher’s exact test.

**Table S6.** Phylogenetic clustering of HIV-1 B_CAR_ and B_PANDEMIC_ *pol* sequences from French Guiana and Suriname.

| Country | B_CAR*_ | | | | B_PANDEMIC**_ | | | |
| --- | --- | --- | --- | --- | --- | --- | --- | --- |
|  | Total | Non-clustered | Small clusters  (*n* < 10) | Large clusters  (*n* > 10) | Total | Non-clustered | Small clusters  (*n* < 10) | Large clusters  (*n* > 10) |
| GF | 162 | 50 (31%) | 17 (10%) | 95 (59%) | 98 | 40 (41%) | 7 (7%) | 51 (52%) |
| SR | 45 | 3 (7%) | 4 (9%) | 38 (84%) | 40 | 6 (15%) | 6 (15%) | 28 (70%) |

* *P* = 0.001 (Fisher’s exact test). ** *P* = 0.17 (Pearson’s Chi2)

**Table S7.** Prevalence of major HIV-1 B_CAR_ lineages circulating in French Guiana and Suriname.

| **Country** | **B_CAR_** | **B_CAR-SA-I_** | **B_CAR-GF/SR-I_** | **B_CAR-GF/SR-II_** | **B_CAR-GF/SR-III_** | ***P**** |
| --- | --- | --- | --- | --- | --- | --- |
| GF | 162 (100%) | 49 (30%) | 20 (12%) | 16 (10%) | 10 (6%) | **<0.001** |
| SR | 45 (100%) | 4 (9%) | 23 (51%) | 9 (20%) | 2 (4%) |  |

*Fisher’s exact test

**Table S8.** Prevalence of major HIV-1 B_PANDEMIC_ lineages circulating in French Guiana and Suriname.

| **Country** | **B_PANDEMIC_** | **B_PAN-GF/SR-I_** | **B_PAN-GF/SR-II_** | **B_PAN-GF-I_** | ***P**** |
| --- | --- | --- | --- | --- | --- |
| GF | 98 (100%) | 33 (34%) | 7 (7%) | 11 (11%) | **0.02** |
| SR | 40 (100%) | 22 (55%) | 6 (15%) | - |  |

*Fisher’s exact test.

**Table S9.** Best fit demographic models for major HIV-1 subtype B lineages circulating in French Guiana and Suriname.

| **Clade** | **Model** | **PS**  **Log ml** | **Models compared** | **Log BF** | **SS**  **Log ml** | **Models compared** | **Log BF** |
| --- | --- | --- | --- | --- | --- | --- | --- |
| B_CAR–SA-I_ | Log | -6842.9 | - | - | -6843.1 | - | - |
|  | Expo | -6850.9 | Log/Expo | 8.0 | -6851.0 | Log/Expo | 7.9 |
|  | Expa | -6853.3 | Log/Expa | 10.4 | -6853.5 | Log/Expa | 10.4 |
| B_CAR–GF/SR-I_ | Log | -5530.8 | - | - | -5531.1 | - | - |
|  | Expo | -5534.0 | Log/Expo | 3.2 | -5534.0 | Log/Expo | 2.9 |
|  | Expa | -5539.0 | Log/Expa | 8.2 | -5539.3 | Log/Expa | 8.2 |
| B_PAN–GF/SR-I_ | Log | -5338.6 | - | - | -5338.8 | - | - |
|  | Expo | -5340.5 | Log/Expo | 1.9 | -5340.7 | Log/Expo | 1.9 |
|  | Expa | -5343.5 | Log/Expa | 4.9 | -5343.8 | Log/Expa | 5.0 |

Log marginal likelihood (ml) estimates for the logistic (Log), exponential (Expo) and expansion (Expa) growth demographic models obtained using the path sampling (PS) and stepping-stone sampling (SS) methods. The Log Bayes factor (BF) is the difference of the Log ml between alternative (H1) and null (H0) models (H1/H0). Log BFs > 3 indicates that model H1 is more strongly supported by the data than model H0.

**Table S10.** Epidemiological information of subjects from French Guiana infected by major HIV-1 B_CAR_ and B_PANDEMIC_ lineages.

| **Characteristic** | **B_CAR–SA-I_**  **(n = 49)** | **B_CAR–GF/SR-I_**  **(n = 20)** | **B_CAR–GF/SR-II_**  **(n = 16)** | **B_PAN–GF/SR-I_**  **(n = 33)** | ***P*** |
| --- | --- | --- | --- | --- | --- |
| **Sampling interval (years)** | 2007-2012 | 2007-2012 | 2007-2012 | 2007-2012 | - |
| **HIV diagnosis***** |  |  |  |  |  |
| 1990-2005 | 6 (12%) | 5 (25%) | 2 (13%) | 2 (6%) | 0.27 |
| 2006-2012 | 43 (88%) | 15 (75%) | 14 (87%) | 31 (94%) |  |
| Unknown | - | - | - | - |  |
| **Age group (years)***** |  |  |  |  |  |
| 18-24 | 3 (6%) | 2 (10%) | - | 2 (6%) | 0.31 |
| 25-34 | 17 (35%) | 7 (35%) | 2 (13%) | 12 (36%) |  |
| 35-44 | 22 (45%) | 6 (30%) | 7 (44%) | 10 (30%) |  |
| >44 | 7 (14%) | 5 (25%) | 7 (44%) | 9 (27%) |  |
| **Sex** |  |  |  |  |  |
| Male | 28 (57%) | 5 (25%) | 10 (63%) | 14 (42%) | 0.054 |
| Female | 21 (43%) | 15 (75%) | 6 (37%) | 19 (58%) |  |
| **Mode of Transmission***** |  |  |  |  |  |
| Homosexual / Bisexual | 1 (2%) | 1 (5%) | 1 (6%) | - | 0.15 |
| Heterosexual | 47 (96%) | 19 (95%) | 13 (81%) | 30 (91%) |  |
| Unknown | 1 (2%) | - | 2 (13%) | 3 (9%) |  |
| **Geographic location***** |  |  |  |  |  |
| Cayenne | 42 (86%) | 7 (35%) | 13 (81%) | 17 (52%) | **<0.001** |
| Saint Laurent du Maroni | 5 (10%) | 13 (65%) | 3 (19%) | 15 (45%) |  |
| Others/Unknown | 2 (4%) | - | - | 1 (3%) |  |
| **Country of birth***** |  |  |  |  |  |
| French Guiana | 6 (12%) | 2 (10%) | 2 (13%) | 6 (18%) | **<0.001** |
| Haiti | - | 1 (5%) | - | 5 (15%) |  |
| Suriname | 5 (10%) | 15 (75%) | 3 (19%) | 13 (39%) |  |
| Guyana | 22 (45%) | - | 1 (6%) | 3 (9%) |  |
| France | 6 (12%) | 2 (10%) | 7 (44%) | 3 (9%) |  |
| Brazil | 8 (16%) | - | 2 (13%) | 2 (6%) |  |
| Others/Unknown | 2 (4%) | - | 1 (6%) | 1 (3%) |  |
| **Clinical Condition***** |  |  |  |  |  |
| Asymptomatic (A) | 38 (78%) | 16 (80%) | 12 (75%) | 26 (79%) | 0.70 |
| Symptomatic (B)/AIDS (C) | 11 (22%) | 4 (20%) | 3 (19%) | 7 (21%) |  |
| Unknown | - | - | 1 (6%) | - |  |
| **Viral load (copies/ml)***** |  |  |  |  |  |
| <LD-10,000 | 28 (57%) | 12 (75%) | 6 (38%) | 6 (18%) | **0.002** |
| >10,000 | 21 (43%) | 8 (25%) | 10 (62%) | 27 (82%) |  |
| **CD4 count (cells/ml)***** |  |  |  |  |  |
| 350-500 | 24 (49%) | 11 (55%) | 8 (50%) | 17 (52%) | 0.98 |
| >500 | 25 (51%) | 9 (45%) | 8 (50%) | 16 (48%) |  |

*Fisher’s exact test. **Pearson’s chi2.
